# Stroke rate detection from accelerometer data on marine mammals

**DOI:** 10.1242/jeb.252217

**Published:** 2026-09-07

**Authors:** Frederik H. Jensen, Outi M. Tervo, Mads Peter Heide-Jørgensen, Susanne Ditlevsen

**Affiliations:** ^1^ Data Science Laboratory, Department of Mathematical Sciences, University of Copenhagen, Universitetsparken 5, DK-2100 Copenhagen, Denmark; ^2^ Department of Mammals and Birds, Greenland Institute of Natural Resources, 3900 Nuuk, Greenland; ^3^ Greenland Institute of Natural Resources, Strandgade 91,2, DK-1401 Copenhagen K, Denmark; ^4^ Technical University of Denmark, National Institute of Aquatic Resources, Section for Ecosystem-based Marine Management, Henrik Dams Allé, Building 201, 2800 Kgs. Lyngby, Denmark

**Keywords:** Tail stroke, Movement, Narwhal, Accelerometer data, ODBA, Signal processing

## Abstract

Overall dynamic body acceleration (ODBA) is widely used as a proxy for movement activity. However, for large, slow-moving animals such as cetaceans, ODBA is dominated by orientational shifts rather than dynamic movements. We argue for using stroke rate frequency as an alternative to ODBA and present a method for estimating stroke rate from acceleration data, using narwhals as a case study. The resulting stroke rates aligned with diving phases and depths, where gliding is expected to be prevalent. The results were also competitive with state-of-the-art magnetometer-assisted approaches. The method also successfully detected individual strokes during a 30 s video of narwhal surface swimming. Using combined surge and heave acceleration produced better stroke estimates than using either in isolation. Our findings demonstrate that accelerometer-based tail stroke frequency is a valid proxy for activity level in marine mammals and highlight the importance of correcting for orientational effects.

## Teaser

**Summary:** A method for improving estimates of marine mammal stroke rates using only accelerometer data, demonstrated through a case study of narwhals.

## INTRODUCTION

Distinguishing between active and passive behavioural states is essential for accurately estimating a species' activity patterns. Firstly, these distinctions can indicate whether an animal is resting or foraging, which is relevant for understanding energy expenditure, foraging strategies and overall behavioural ecology. Secondly, energy is often a resource that must be carefully conserved, and overexposure to stressful situations can harm a species' overall fitness. Estimation of activity and energy expenditure is especially relevant for deep-diving ocean life, such as marine mammals. For most marine mammals, their behaviour is difficult to observe when in deep waters and in remote and inaccessible areas. Increasing anthropogenic activity and climate change are known to be potential stress factors that affect the activity levels of marine predators.

In this study, we used data from the narwhal (*Monodon monoceros*) as a case study for detecting activity in marine mammals. Because of their elusive nature and extreme habitat selection, documentation of narwhal behaviour remains incomplete. Furthermore, previous research has shown that narwhals are highly sensitive to noise, showing behavioural changes when under the influence of an anthropogenic sound source up to 20–40 km away (Heide-Jørgensen et al., 2021). Estimating activity levels can indicate to what degree the narwhal's energy balance is disturbed when under stress. When observation of animal activity is not feasible, it must be inferred indirectly. For this purpose, accelerometer tags are invaluable (Brown et al., 2013). Recordings of acceleration tags consist of high-frequency moment-to-moment movement and orientation, which have proven effective in recognising behavioural patterns in a wide range of free-ranging animals, including aquatic, terrestrial and avian species (Sakamoto et al., 2009; Conners et al., 2021; Clarke et al., 2021). For narwhals specifically, accelerometer data have been used to detect foraging times during deep dives (Ngô et al., 2021; Jensen et al., 2023).

One of the most commonly used acceleration-based activity estimators is overall dynamic body acceleration or ODBA (Wilson et al., 2006). ODBA is the sum of the absolute acceleration in all three directions after correcting for gravitational pull. Vectorial dynamic body acceleration (VeDBA) is sometimes suggested as an alternative to ODBA as animals accelerate along a vector rather than along three straight paths (Qasem et al., 2012). Both ODBA and VeDBA assume that orientational effects on acceleration will change slowly (Gleiss et al., 2010). However, this assumption is not always reasonable, as animals often make cyclical realignments when active.

For example, marine mammals rely on ventro-dorsal tail strokes to swim. These strokes will induce locomotion, as well as up and downward changes in pitch at the same frequency. These two effects are inseparable without additional information on the animal's heading. This means that the magnitude of acceleration from a fluke stroke will be the sum of the projection from muscle contractions and the change in the direction of gravitational pull. Of these, only muscle projection is directly related to energy expenditure in a meaningful way. We refer to these different forces using the terminology of Martín López et al. (2022). The filtered acceleration used for calculating ODBA is referred to as dynamic acceleration. Dynamic acceleration will be the sum of short-term alignment-related acceleration referred to as dynamic orientation acceleration (DOA) and locomotive effects from muscle contractions (specific acceleration). The long-term orientational effects separate from the dynamic acceleration are referred to as static acceleration. Whether DOA overshadows specific acceleration depends on various factors. The magnitude of specific acceleration from a tail stroke will increase with the stroke rate and body displacement (Johnson, 2011). In contrast, neither frequency nor displacement affects the angular orientational effects of DOA. Consequently, high-frequency movements with large displacements will result in a larger contribution from specific acceleration to overall dynamic acceleration. Tag placement will also have an effect, as the body displacement decreases with the distance from the movement. For tail strokes, the body displacement from tail strokes will be larger the closer the tag is to the tail (Johnson, 2011).

As movement frequency scales with the inverse of body length (Sato et al., 2006), the specific acceleration will be proportionally greater than the DOA for smaller animals (for a given tag placement). The effect is somewhat offset by larger animal movements having larger body displacements (Martín López et al., 2022). Martín López et al. (2022) found that ODBA yielded the best results for estimating physical activity in smaller animals. For larger animals over 3 m, the ODBA measurements increasingly represented changes in alignment. Specifically, ODBA had a similar magnitude of specific and orientational acceleration for Blainville's beaked whales (*Mesoplodon densirostris*), while orientational effects dominated ODBA for sperm whales (*Physeter macrocephalus*). Narwhals are slow moving, and of similar size to beaked whales. In addition, narwhals are prone to rolling (Dietz et al., 2007). Rolling will induce strong ODBA measurements from the short-term gravitational shifts. As a result, ODBA has a tendency to overestimate the effort of rolling, especially as the narwhal, among other marine mammals, has dorsal ridges instead of fins. A generalised method for recognising marine mammal activity should, therefore, ideally accommodate this variation. Narwhals also have higher neck flexibility compared with most other cetaceans due to their unfused cervical vertebrae. This could also generate misleading ODBA values if the tag is attached too close to the head.

**Fig. 1.**
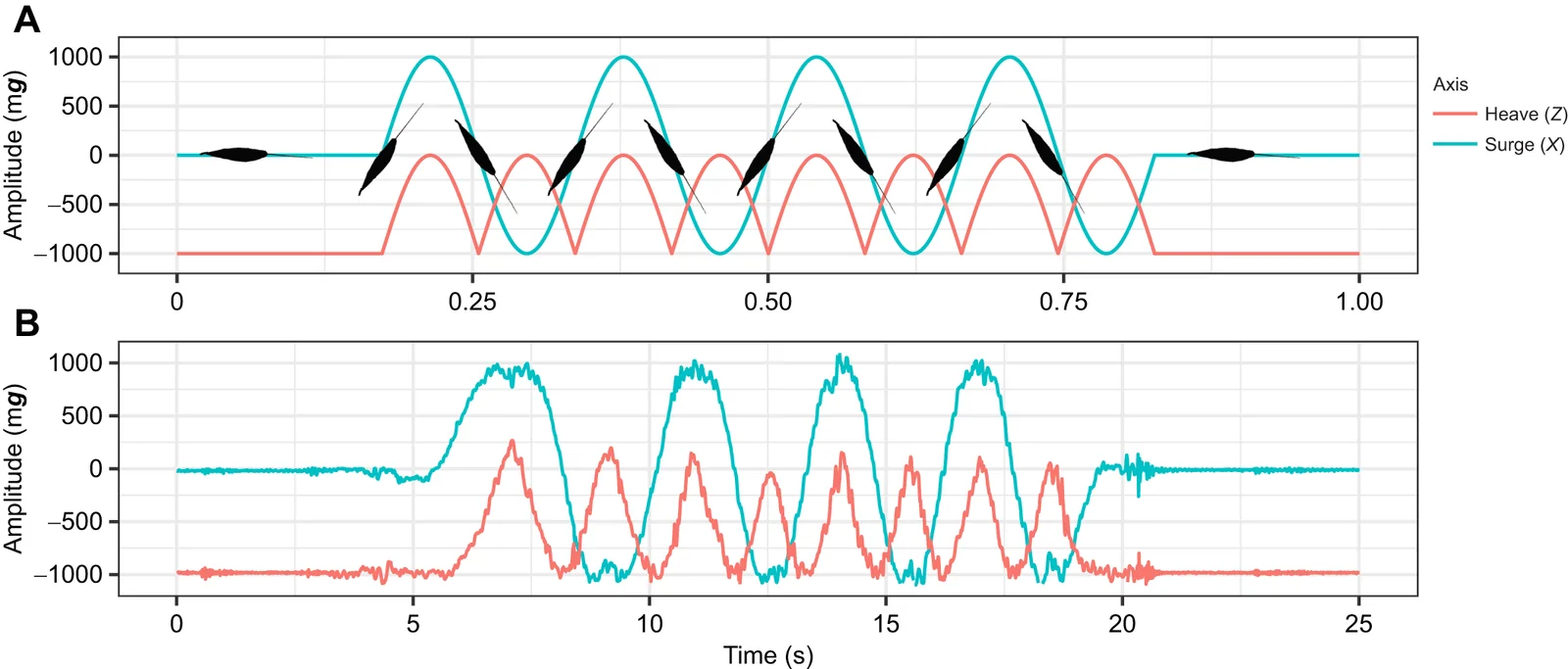
**Amplitude of acceleration data in heave and surge directions.** (A) A simulated acceleration signal from an upright tag being pitched up and down by 90 deg. As shown, the *Z* signal will be double the frequency and half the amplitude. (B) Acceleration signal from a tag being manually pitched up and down in the same setting.

An alternative to ODBA is to estimate physical activity from the frequency of movement cycles. For marine mammals, this would be the rate at which the animal strokes its tail. Stroke frequency has been used to indicate behavioural activity in a wide range of sea life, such as seabirds (Sato et al., 2008), Greenland sharks (Watanabe et al., 2012) and various seals and whales (Williams et al., 2000; Sato et al., 2003). In marine mammals, the fluke stroke rate increases linearly with swimming speed (Williams et al., 2017), which itself is closely related to activity level. Fluke stroke frequency has the benefit of being largely invariant to positional changes. However, the amplitude will still be sensitive to orientational dynamics. Dorsoventral strokes (e.g. birds and marine mammals) will induce pitching oscillations on acceleration, while caudal strokes (e.g. most fish) induce sway oscillations. As a result, we can still derive the stroke rate periodicity from the gravitational shifts in the pitch direction even when the dynamic acceleration is dominated by DOA. However, the magnitude of the strokes can potentially be misleading as DOA is sensitive to gimbal lock. Pitching motions also depend on alignment. If a tag is heading straight ahead and pitches up and down at 90 deg angles, the gravitational pull will shift from towards to away from the Earth's centre in the surge direction, but shift around the centre in the heave direction. As a result, the heave signal will have double the frequency and half the amplitude of the surge signal. The opposite will be the case if the animal is headed straight up or down. If unaccounted for, these dynamics can bias stroke rate estimates, making the animal appear to stroke at twice the true rate or not at all. The effects are illustrated in Fig. 1, where the dynamics are simulated and recreated by manually pitching an unattached tag up and down.

Early cetacean fluke detection was done by counting oscillations in pitch (Nowacek et al., 2001; Miller et al., 2004; Johnson and Tyack, 2003; Simon et al., 2009). Pitch angles are fairly straightforward to estimate from accelerometer data, but do not account for the aforementioned orientational effects. The approach is, therefore, suboptimal for cases where DOA dominates the acceleration signal, and orientational shifts when swimming are common.

To account for orientation, Simon et al. (2012) presented an approach that derives a direction cosine matrix from acceleration and magnetometer data to rotate the pitch signal. Flukes estimated from the corrected pitch in the sagittal plane will be irrespective of posture. Magnetometer data only record directional shifts with respect to the magnetic poles, whereas accelerometer data include effects of specific movements. This approach was also included in a study on Bryde's whale feeding tactics (Izadi et al., 2022). A later approach approximated short-term orientational shifts in pitch from a rotational matrix obtained from filtered magnetometer data (Martín López et al., 2015). Specific acceleration could then be estimated by subtracting the orientational effects from the corresponding filtered accelerometer data. Movements were also quantified exclusively from magnetometer data in Shepard et al. (2008), where the benefits and challenges associated with magnetometer data were also explored in greater detail.

Body rotations can also be obtained from gyroscopes. Gyroscopes can estimate rotations in all three axes and are well suited for estimating complex and rapid movements. This comes at the cost of higher power consumption and calibration sensitivity (Martín López et al., 2016). Gyroscope oscillations have been used to quantify how tail stroke frequency and cruising speed scale with body length Gough et al. (2019).

Stroke estimation relying solely on acceleration data usually defaults back to using either pitch angle or one-dimensional acceleration. As an example, both Martín López et al. (2015) and Gough et al. (2019) have data series where only acceleration was available because of tag specifications and malfunctions, respectively. In these cases, fluking was estimated exclusively from acceleration in the heave direction. Likewise, in papers where fluking was estimated exclusively from accelerometer data, acceleration was only considered in one dimension (Williams et al., 2017; Tervo et al., 2021). This is not necessarily a problem for smaller or fast-moving animals, as the dynamic acceleration will be dominated by specific acceleration. If the animal's alignment is fairly constant during movement, the orientational effects will also be negligible. However, we argue that neither assumption is reasonable for the narwhal.

In this paper, we suggest an approach for estimating tail strokes purely from accelerometer data. We then present an approach to estimate stroke frequency that accounts for vertical heading during dives, and demonstrate it using narwhal tagging data. This method is compared with the magnetometer-assisted approach of Martín López et al. (2016) to assess whether proper estimations can be obtained exclusively from acceleration data.

## MATERIALS AND METHODS

### Data acquisition

**Fig. 2.**
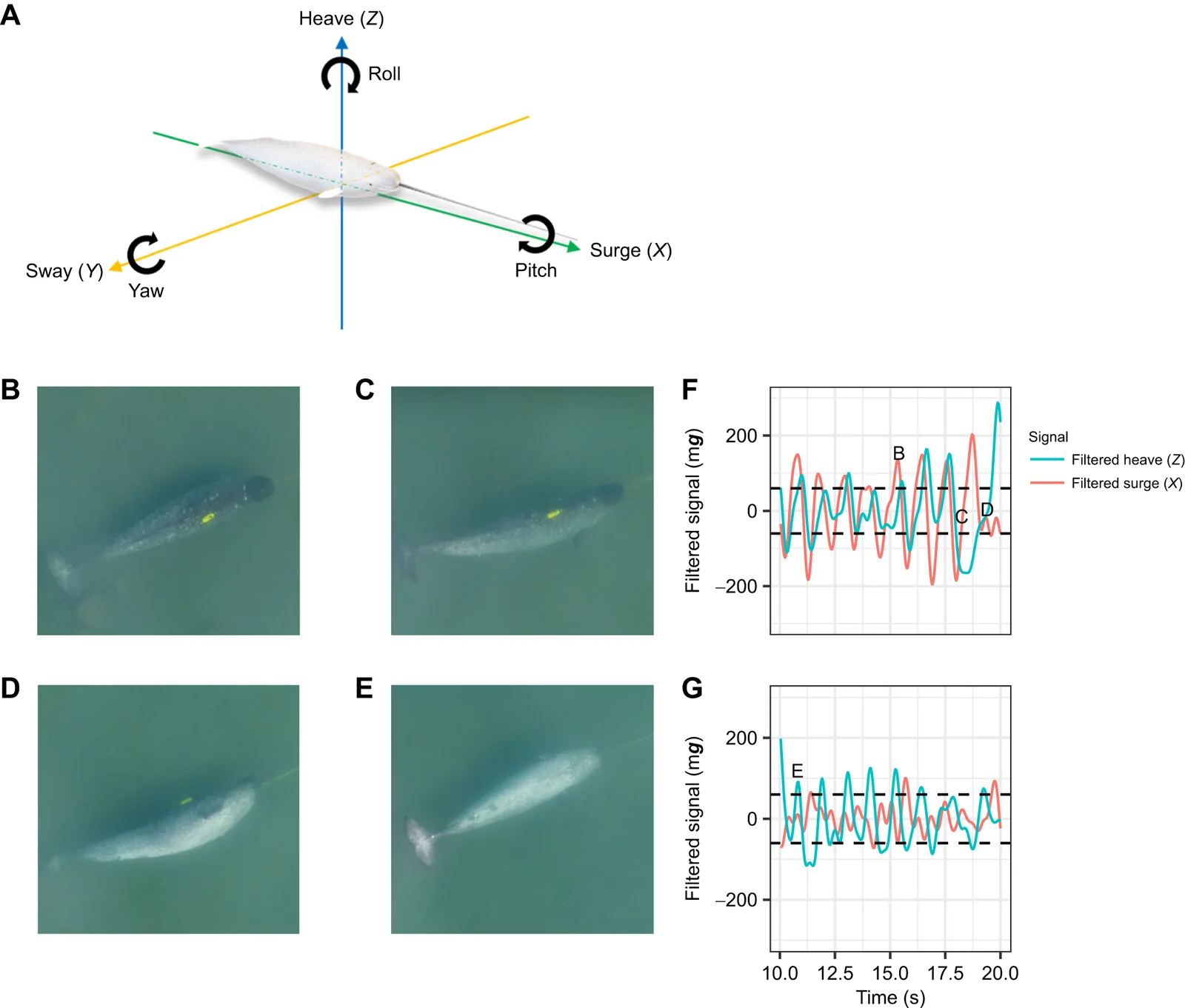
**Drone footage of a narwhal and associated acceleration data.** (A) A visual representation of how surge, sway and heave relate to a narwhal with an accelerometer tag attached to its dorsal ridge. Rotational movements about the three axes (pitch, yaw and roll) are also shown. (B–E) Pictures of male narwhal 2018MM7 before, during and after rolling on its back. (F,G) Filtered surge and heave acceleration from the first 20 s of the video. Letters mark when pictures B–E were taken. Dashed lines indicate selected upper and lower thresholds for stroke amplitude.

Data were recorded from 10 narwhals, *Monodon monoceros* Linnaeus 1758 (8 males and 2 females), caught in the late summer period of 2016–2018. One male whale was tagged both in 2017 and in 2018 (2017MM3 and 2018MM3). Each time series spanned 1–6 days. To account for unusual behaviour after tagging and release, data before the first vocalisation were discarded (Blackwell et al., 2018). The narwhals captured in 2018 were part of an exposure study (Heide-Jørgensen et al., 2021). For these narwhals, only data before exposure were included in the analysis to avoid confounding effects from atypical behaviour. The whales were captured in collaboration with local Inuit hunters and equipped with Acousonde acoustic and orientation tags (www.acousonde.com) before they were released back into the wild. Tags were fastened to the dorsal ridge and reinforced with a magnesium corrodible link and 1 mm nylon thread to extend recording durations. The tagging and data collection process is described in greater detail elsewhere (Blackwell et al., 2018; Heide-Jørgensen et al., 2015). An overview of narwhal biomarkers and tagging periods for each time series can be found in figure 1 of Jensen et al. (2023). The study was reviewed and approved by the Institutional Animal Care and Use Committee of the University of Copenhagen (17 June 2015). Permission for capturing, handling and tagging of narwhals was provided by the Government of Greenland (Case ID 2010±035453, document number 429 926). Each data series consists of depth measured in metres at 1 Hz and acceleration measured along three axes, denoted *X*, *Y* and *Z*. Positive samples along the *X*-axis correspond to the animal pointing up, while positive samples along *Y* and *Z* correspond to being left-side up and upside down, respectively. The *X*, *Y* and *Z* directions correspond to surge, sway and heave. Data also include approximately 30 s of data from a male narwhal immediately after release from capture in 2018 (2018MM7) (Williams et al., 2022). These data include drone footage, allowing for visual comparisons of the tail strokes. Pictures from the footage are displayed in Fig. 2B–E. A visual representation of the triaxial directions with respect to the narwhal heading can be seen in Fig. 2A. Here, only the two main rotational axes are relevant for the analysis. The *X*-axis measures pitching when rotating around the *Y*-axis. Ventro-dorsal swimming will be visible here. The *Z*-axis measures rolling when rotating around the *X*-axis. This axis will determine how affected the signal is by gimbal lock.

Acceleration was measured at 100 Hz, but downsampled to 25 Hz in the interests of storage, as higher frequencies are low-pass filtered in the data processing anyway. Dives were partitioned into four phases: surface, descending, bottom and ascending, following the procedure in Tervo et al. (2021). For comparing methods, magnetometer data were included for two narwhals (2016MM3 and 2018MM2). These data were sampled at 10 Hz.

### Filtering of the acceleration signal

The acceleration signal was initially band-pass-filtered to separate static acceleration and high-frequency noise. The cut-off frequency for the low-pass filter was set to half the dominant signal frequency (Martín López et al., 2022). For the narwhal data, the dominant signal frequency was determined by analysing data shortly after release. The stroke signal is strong during escape, as the narwhal is actively swimming with constant heading, shallow diving and no rolling. In particular, the female whale (2016MM3) showed a long period, approximately 3 h after release, with shallow diving, which was used for determining the dominant signal frequency. According to Martín López et al. (2015), surge acceleration is proportional to heave, but often equal to or less than that during steady cetacean swimming. *Z* was therefore chosen for determining the dominant signal frequency. However, *X* indicates a similar frequency.

**Fig. 3.**
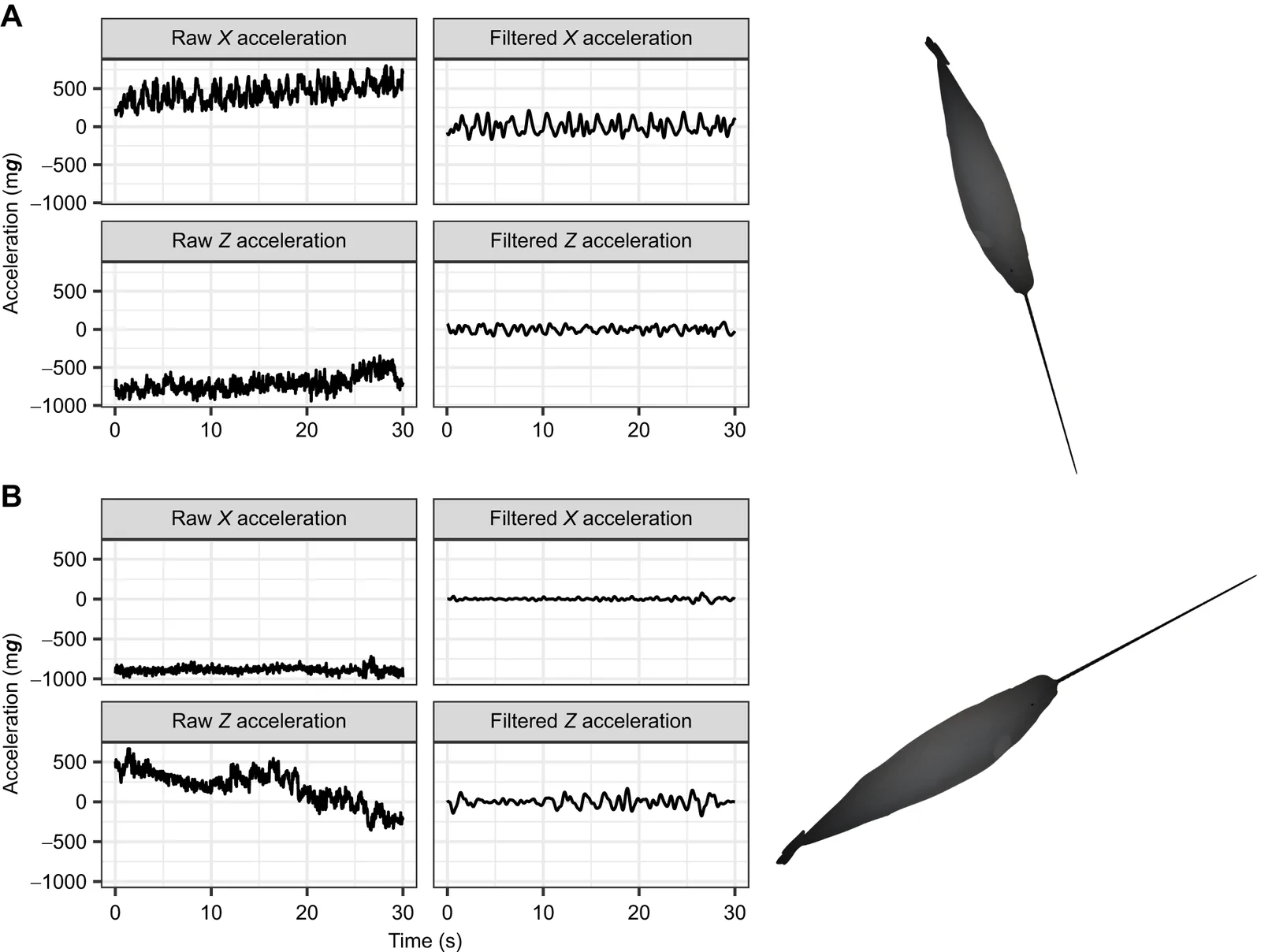
**Raw and filtered acceleration signal in the *X* and *Z* directions for two periods.** (A) Period with surface swimming at a slight upwards angle. (B) Period of deep-dive descent. Narwhal silhouettes to the right of the plots show approximate alignment during the two periods.

A smoothed periodogram was generated for the *Z* acceleration using fast Fourier transformation in Fig. S1A, which indicates the dominant signal frequencies. The plot shows a peak in the lowest frequencies, probably stemming from orientational effects, but also a second peak around 0.52 Hz, which corresponds to an expected stroking rate of approximately one stroke every other second. The resulting high-pass cutoff is roughly 0.26 Hz. To reduce noise, a low-pass filter was also applied to the acceleration signal with a cutoff of 2 Hz, as it was deemed unrealistic that narwhals can stroke faster than 2 tail strokes per second. Both high- and low-pass filters were implemented using a Butterworth filter (Butterworth, 1930). The low-frequency acceleration separated by the high-pass filter estimates the static acceleration, which was used to approximate alignment in the form of pitch and roll angles. The procedure for estimating alignment from gravitational effects is described in Shepard et al. (2008). Raw and filtered acceleration for selected data excerpts can be seen in Fig. 3.

### Stroke discretisation

To classify a stroke, a minimum amplitude threshold was applied to the filtered signal. As the filtered acceleration signal is largely symmetric around zero, the same amplitude magnitude was used for the lower and upper thresholds. The specific value of the threshold was determined by evaluating the resulting stroking rates over a range of potential thresholds. The threshold should be set sufficiently low to detect actual strokes, but high enough that the double-frequency, half-amplitude signal from stroking around the direction of gravity is not counted as two strokes.

**Fig. 4.**
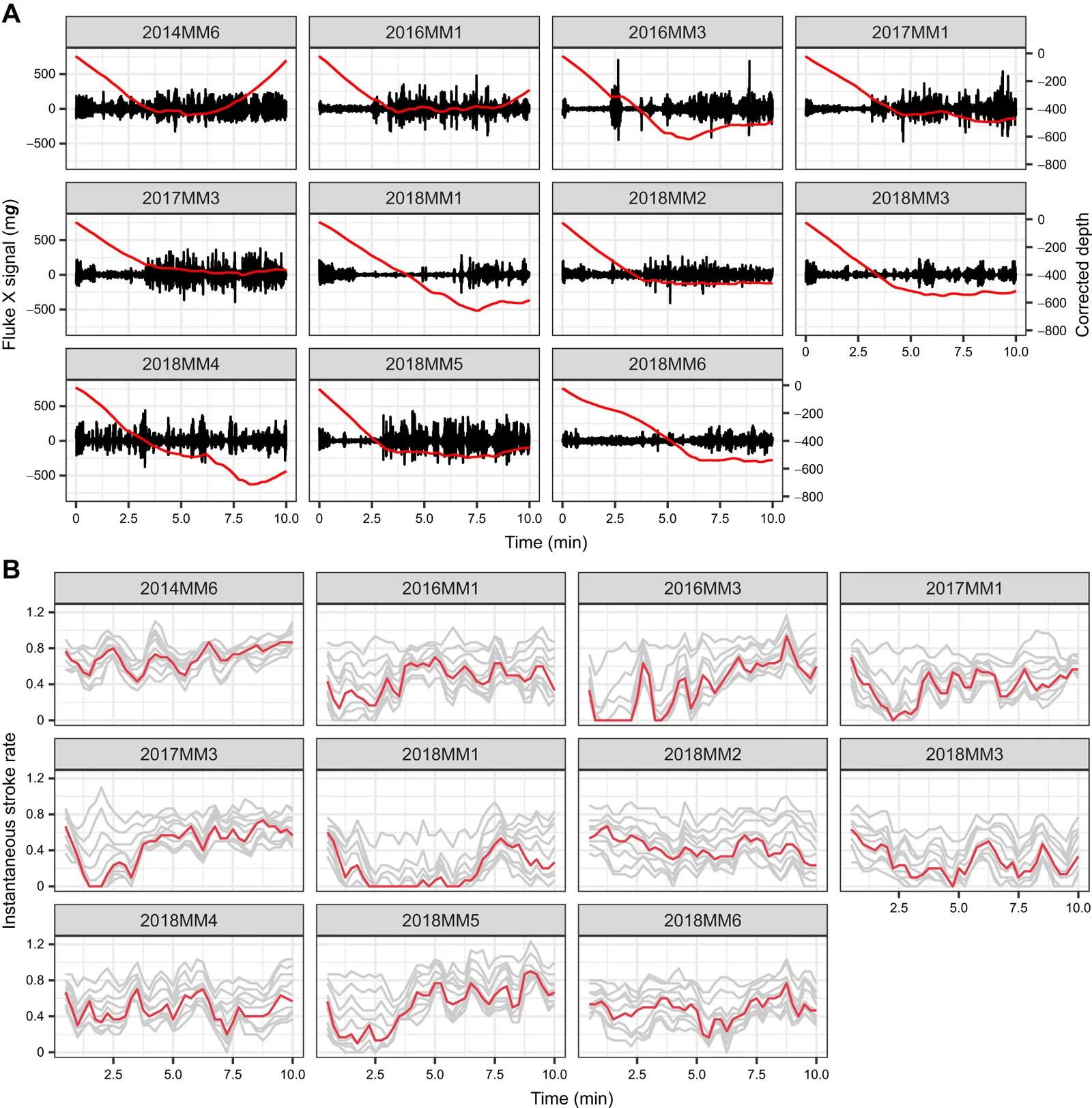
**Stroke rate for different amplitude thresholds from each narwhal.** (A) Filtered *X* acceleration. The red line indicates the simultaneous corrected depth on the secondary axis. (B) The instantaneous stroke rate in 20 s intervals for various stroke thresholds. Lower threshold yields higher stroking. The chosen threshold of 60 m***g*** is marked in red.

As argued, orientation affects which axes best capture tail stroking. This is also apparent from the data excerpts in Fig. 3. In Fig. 3A, the approximate (roughly straight swimming) stroke signal is clearest in the filtered *X* acceleration data, whereas in Fig. 3B (descent swimming), strokes are clearer in the filtered *Z* acceleration. Strokes are also visible in the raw acceleration only on the corresponding axis. We suggest a simple approach for combining the two axes. Using the filtered acceleration signals, strokes are determined by upcrossings in amplitude that passed over the band threshold as displayed in Fig. 2F,G. The selected threshold was ±60 m***g*** as presented in Fig. 4. From the data, surge and heave did not show any differences in amplitude. As a result, the same threshold for defining a stroke was used for both axes. Again, heave acceleration on the *Z*-axis was used as the main axis for initially counting strokes (Martín López et al., 2015). Strokes from the *X*-axis were then subsequently added to the total stroke count. To avoid double-counting strokes that induce signals on both axes, *X*-axis strokes were only added to the total stroke count if they did not occur within 2 s of a stroke on the *Z*-axis. The 2 s overlap was determined according to Fig. S1A, which shows a dominant stroke frequency of approximately one stroke every other second. If multiple *X*-axis strokes occurred within 2 s of a stroke on the *Z*-axis, only the first was discarded to prevent over-correction. The entire procedure is also presented in the algorithm (see Box 1). The results of the procedure were compared with the magnetometer-assisted approach of Martín López et al. (2015). To avoid confounding effects on comparisons, we chose to apply the same filtering procedure to the magnetometer data as to the accelerometer data.

Box 1. Algorithm 1 – stroke detection procedure The stroke detection procedure was as follows:

(1) Filter *X* and *Z* acceleration data using an appropriate band-pass range. We suggest half the dominant stroke frequency cutoff.
(2) Count upcrossings above the selected thresholds in the filtered *X* and *Z* signals. Register each stroke at the time of maximum amplitude between successive upcrossings.
(3) *Z* is chosen as the baseline axis and used for initial stroke counts.
(4) If an *X*-stroke is detected within 2 s of a *Z*-stroke, it is assumed to originate from the same stroke event and is counted once at the time of the *Z*-stroke. If two *X*-strokes occur within 2 s of a single *Z*-stroke, only the first *X*-stroke is discarded.
(5) Summarise activity level as strokes per unit time over a window defined by variance in stroke rate across consecutive windows.

To quantify activity levels, stroking was evaluated in an appropriate window. We define instantaneous stroking rate (ISR) as the number of strokes divided by the window length in seconds. Window length should be large enough to smooth individual strokes, yet small enough to avoid averaging out different behavioural states. In Fig. S1B, the ISR can be seen for various window lengths in a 10 min data excerpt. The lower window lengths of approximately 10 s show strong fluctuations in ISR. When the window length increases, the fluctuations lessen and the ISR curve becomes smoother. Fig. S1C shows the total variance in incremental differences for various window lengths. The elbow point of this curve was used to determine an appropriate window length, as this point shows where the initial fluctuations have been averaged out. The exact window length chosen was 20 s. As DOA amplitude decreases as the narwhal's orientation approaches a sideways position, we examined the amount of time and duration of events in which the narwhals swam within ±30 deg of a lateral position and whether side-swimming should be accounted for.

## RESULTS AND DISCUSSION

The 20 s interval stroking rate for different amplitude thresholds is shown for selected 10 min data segments from each narwhal in Fig. 4. Candidates for threshold value were chosen in a range between 10 and 100 m***g*** in increments of 10 m***g***. The lines with a higher stroke rate correspond to having a lower threshold and vice versa. Segments were chosen from dives of at least 400 m in depth. For the lower thresholds, the stroke rate remained high throughout, despite the acceleration signals showing varying activity levels. The trend is most obvious for 2018MM1, where an extended period with low amplitude still resulted in a stroke rate of approximately 0.5, or one stroke every other second. Conversely, the higher thresholds seem to estimate too little activity. In particular, towards the end of the records of 2018MM1 and 2018MM2, the lower lines indicate stroke rates close to zero, though the acceleration plot above indicates some activity. This suggests that thresholds too close to 10 or 100 are incompatible with the data. Otherwise, there does not seem to be a clear choice for an optimal threshold. Probably, narwhals make smaller stroke-like movements, even when gliding. What constitutes a stroke is also less discernible in comparison to, for instance, a human step, which can be identified by lifting and placing a foot. We selected 60 m***g*** as an appropriate middle ground, yielding stroke rates broadly consistent with previous results (Tervo et al., 2021). We reasoned that as long as the relative stroking activity is the same across different dive phases and specimens for a given threshold, the specific threshold choice is not important for estimating relative activity levels. The selected threshold is highlighted in the plots.

However, as energy expenditure increases non-linearly with stroking frequency, the specific energy consumption will be influenced by the chosen threshold. For this study, we made no distinctions between strong or weak amplitude strokes. This means that if the narwhals made more vigorous strokes while keeping the frequency somewhat constant, the increased activity would not be captured by our estimations. As argued, separating orientational and specific acceleration is impossible without additional data sources such as gyroscopes or magnetometer data. Because we believe the orientational effects dominate the specific acceleration for larger animals, the strength in amplitude is more likely to be due to positional variation and pitch angle, which do not necessarily correspond to higher activity. To correct for variation in amplitude, we believe that data sources, such as magnetometers or gyroscopes, are necessary. Nonetheless, for estimating energy expenditure, one should consider reducing the expected stroke energy consumption when using a low-amplitude threshold for strokes.

**Fig. 5.**
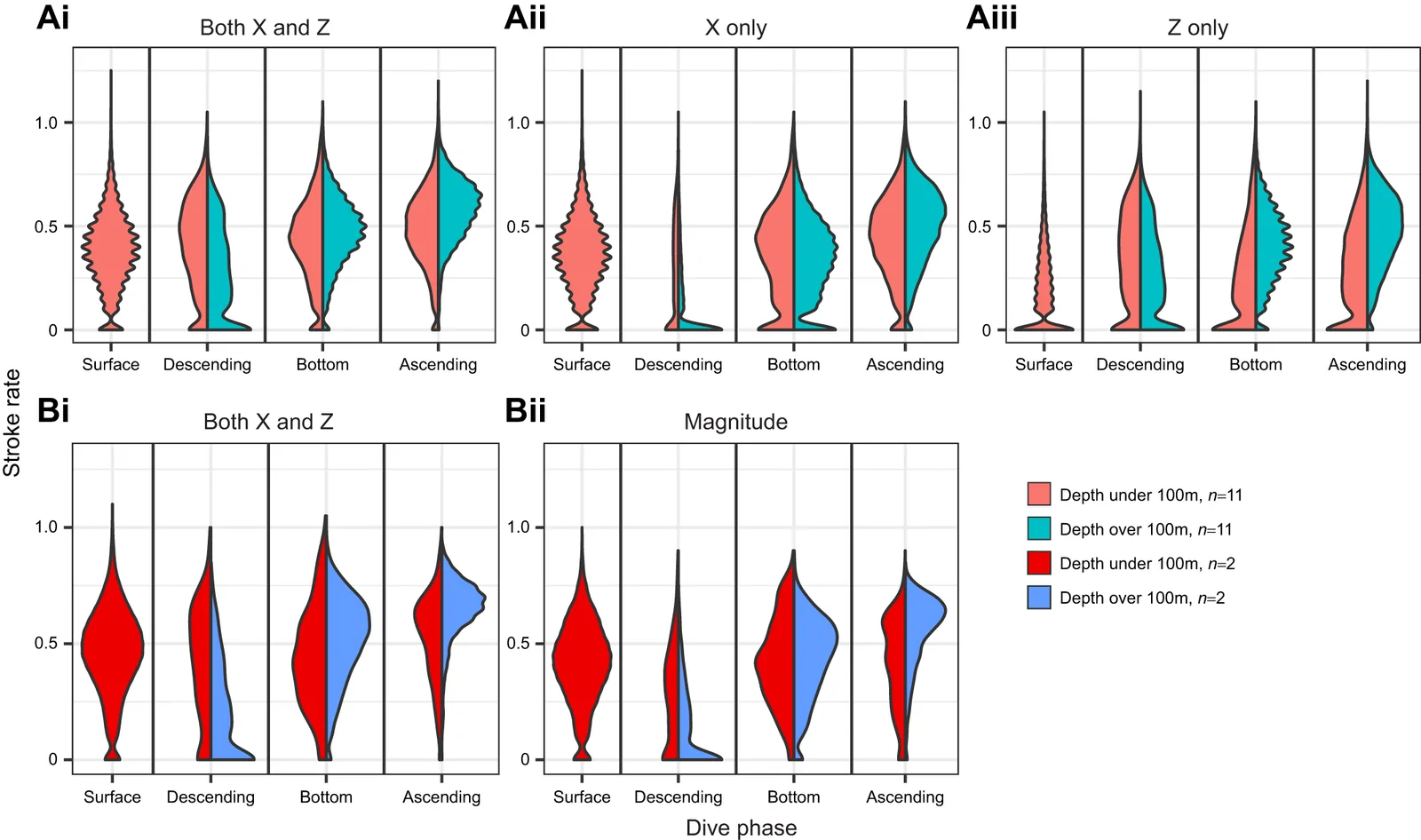
**Split violin plots showing estimated stroke rates across different dive phases and depth categories.** (A) Stroke rates estimated using (i) combined *X*- and *Z*-axis acceleration, (ii) *X*-axis acceleration only and (iii) *Z*-axis acceleration only, for all tagged narwhals (*n*=11). (B) Comparison of stroke rate estimates for two individuals (2016MM3 and 2018MM2; *n*=2) obtained using (i) combined *X*- and *Z*-axis acceleration and (ii) the magnetometer-assisted approach. Data are for surface, descending, bottom and ascending dive phases and <100 m (red) and >100 m (blue) depth categories.

For the selected threshold and 20 s intervals, stroke rate is shown for different dive phases in Fig. 5 across all time series. For comparison, the stroke rate is shown when derived using only *X* acceleration, only *Z* acceleration and both acceleration signals. To account for buoyancy, swimming was partitioned into shallow and deep categories. The exact depths at which narwhals become negatively buoyant are unknown, but for other cetaceans, this occurs at shallower depths than 100 m (Miller et al., 2004; Nowacek et al., 2001). Evidence suggests that the narwhal becomes negatively buoyant at similar depths (Tervo et al., 2021). Categories were determined by depth at a given time and not the target depth of a dive.

Active swimming is required during descent when buoyant and ascent when sinking. This trend was consistent across all estimated stroke rates: in the deep category, stroke rates were lower during descent and higher during ascent compared with the shallow category. The discrepancy was largest for *X* during descent and *X*+*Z* during ascent. For *X* in particular, the deep descending phase was heavily centred on gliding. The deep bottom dive also had a noticeable period of gliding. If the narwhal descends at a straight down angle, it may be that the stroke signal on *X* is not registered because of the gravitational dynamics, whereas the signal is detected when including *Z*. Another possibility is that the narwhal is rolling at the same frequency as the tail strokes, which would induce a periodic signal on *Z*, but not *X*. The stroke rates derived from *Z* and *X*+*Z* also had a lot of gliding compared with other deep phases, but were more skewed towards a broad range of stroke levels.

For the stroke rates derived from *Z*, the distributions were skewed lower across all phases at shallow depths. In comparison to the *X* stroke rates, the deep bottom and descending phases had fewer periods with stroke rates of zero. This agrees with our expectations, as narwhals will swim in a more upright position during shallow dives and surface times, where stroke amplitude is the most visible on *X*. For the deep ascending phase, the stroke rate using both *X* and *Z* had almost its entire density distributed above 0.25 whereas using *X* and *Z* in isolation gave stroke rates down to 0. This result is of interest, as deep dive ascent is generally driven by the need to resurface for air. Therefore, one would expect this phase to have less gliding or none at all. Any gliding during ascent is expected to occur while the whale is buoyant (Nowacek et al., 2001), as this allows it to conserve energy while naturally ascending toward the surface. This could explain the overall lower stroke rates at shallow ascent phases and supports the hypothesis that narwhals stop being buoyant at depths around 100 m. The plot in Fig. 5B also shows the combined *X* and *Z* acceleration stroke rates compared with the magnetometer-driven approach (Martín López et al., 2015) for 2016MM3 and 2018MM2. The results are, more or less, consistent between the magnetometer and accelerometer approach, although the stroke rate distribution was centred slightly lower for the magnetometer method across all phases on a scale of 0.1–0.2 strokes per second. The difference is most visible for the shallow descending phase. Compared with the stroke rates derived from either *X* or *Z*, there seem to be no outlying dive phases, such as the *Z* surface phase and *X* descent phase. Likewise, there is still a complete lack of gliding in the deep ascent phases just as before. The lower stroke rate for the magnetometer approach could be due to the two methods using different amplitude thresholds for constituting a stroke. Another possibility could be that strokes with weak pitching motions and strong locomotion are only detected by the acceleration data. Overall, we deem that our acceleration-based implementation seems to provide competitive estimates without the need for magnetometer data as the general tendencies in activity are the same across the two methods.

The acceleration filtration and amplitude threshold were also applied to the short dataset, where visuals of a narwhal were available (Movie 1 and Fig. 2). The tail strokes from the video show a corresponding oscillation in the filtered acceleration. Initially, the signal was strongest in the *X* direction (Fig. 2F), probably due to the straight heading. After approximately 10 s, the narwhal rolls onto its back and swims down at an angle. Here, the signal becomes stronger on *Z* as expected (Fig. 2G). Key poses from the roll are shown in Fig. 2B–E. Stroke amplitude ranged from well above to slightly below the selected threshold, again indicating no clear evidence for threshold choice. The strokes before the roll seem to have a slightly higher amplitude than before, probably due to the descending swimming angle after the roll. The stroke amplitude varied from being far stronger than the threshold to being slightly too weak to be detected. There was no obvious visual distinction between the high- and low-amplitude strokes. For some of the weaker tail movements, it is unclear from the footage whether they should be considered a stroke or gliding. Overall, the chosen threshold seems to be appropriate for this short data series, although the lower bound falls slightly short at times after the roll. As in the previous results, the signal was strongest along the *X*-axis before rolling but shifted to *Z* when the narwhal started diving.

The placement of the tag will have an effect on the strength of the stroke signal. The tag displacement from strokes will be stronger when the tag is attached close to the tail. However, alignment estimates will be more inaccurate. As tagging experiments are often shared across studies, estimating stroke frequency remains valuable even when tags are not deployed for that purpose.

In total, 9.6% of the data relate to time spent swimming on the side. Further analysis revealed that the narwhal only swam on the side momentarily, probably as part of a roll. Side-swimming events lasting longer than 10 s accounted for approximately 19.6 h out of the total of 25.7 days of recording, which corresponds to approximately 3.2% of the data. For events longer than 20 s, the total drops to 4.9 h. Side-swimming is therefore expected to have a minimal impact on our estimates. Visualisations of side-swimming prevalence and duration are available in Fig. S2. Still, the method could be improved to better account for side-swimming. We tested scaling the signal amplitude using various sigmoid functions of the rolling angle, but the results were found to be unreliable.

While some challenges persist in estimating activity from accelerometer data, there is no universal solution besides observing the tail strokes directly. Biologging marine mammals often involves invasive methods such as capture/release of potentially vulnerable species. It is, therefore, important not only to aim for accurate behavioural estimations but also to use methods that maximise output from tagging. Acceleration and depth data are inexpensive in terms of both data storage and power consumption, at least in comparison to other biologging data types. In this regard, the estimated stroke frequency has a lot of uses for storage-efficient behaviour estimations from longer-term tagging. This can help to further knowledge of the species and their well being, such as by determining energy expenditure to food intake or stress reactions from exposure. Tail stroke estimations could also potentially be on-board processed and relayed by satellite to allow for even longer tagging periods. For narwhals on the Greenland east coast specifically, this could help reveal behavioural patterns during winter, where they are too far from shore to be captured and tagged. Data from these periods, therefore, rely on long tagging periods. The addition of gyroscopes and, to a lesser extent, magnetometer data requires more advanced tags and increases power consumption, as multiple data streams must be stored and processed. It also increases the risk of data gaps if one data source is found missing or unreliable. We believe a simple stroke approximation relying solely on acceleration would be beneficial in this regard.

## Supplementary Material

10.1242/jexbio.252217_sup1 Supplementary information
