# Supplementary material for: Stroke rate detection from accelerometer data on marine mammals
Source: J Exp Biol. 2026 Sep 7;229(17):jeb252217. doi: 10.1242/jeb.252217 (PMC13615681; doi:10.1242/jeb.252217)
Supplement: Supplementary file 1 [file jexbio-229-252217-s1.pdf]

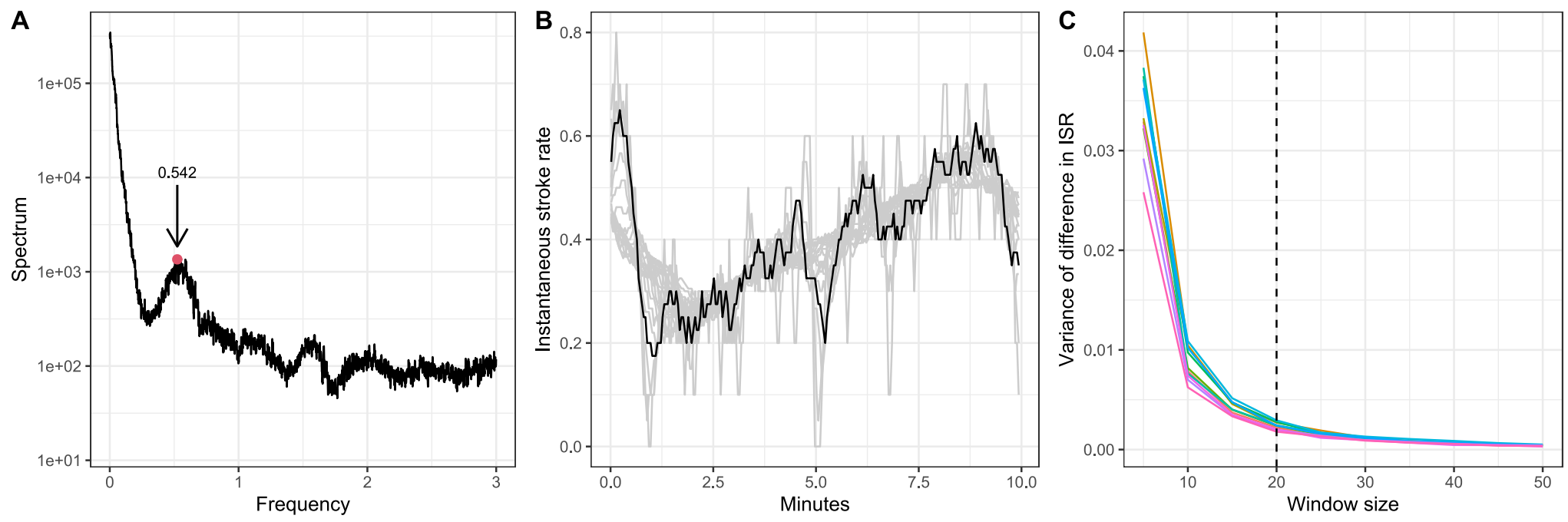

Fig. S1. A: Smoothed periodogram of raw Z acceleration from period with active surface swimming from a female narwhal (2016MM3) showing dominant frequencies of acceleration oscillations with a second peak at *0.52* Hz. B: 10-minute data excerpt showing instantaneous stroke rate estimated over various window sizes, ranging from 10 to 200 seconds. The chosen window of 20 seconds is highlighted in black. Data is from a female whale (2016MM3). C: Total variance in incremental change in ISR as a function of window size for all 11 time series, used to identify an appropriate window frame to describe activity in. The window size should be selected so that incremental time frames do not vary greatly in activity level and are sufficiently smooth. This is done by detecting the point at which the variance drop-off from increasing window size becomes marginal. Based on the plot, a 20-second window was chosen and marked with a dashed line.

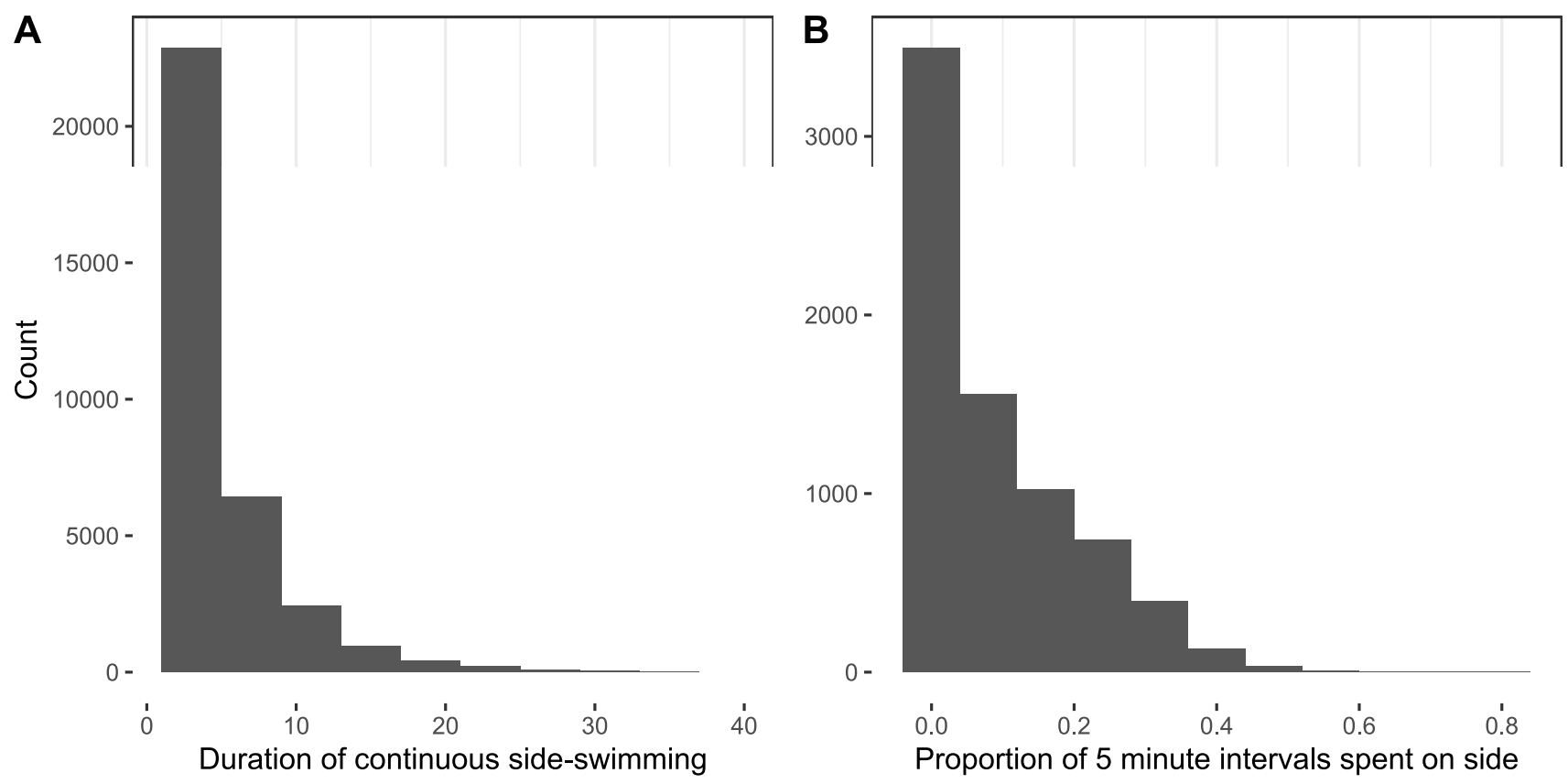

Fig. S2. A: Histogram showing distribution for proportion of 5-minute intervals spent swimming on the side across all narwhals. B: Histogram of distribution of recorded side-swimming durations. There was a marginal number of events of side-swimming lasting longer than 40 seconds, but they were excluded from the plot for better visibility.

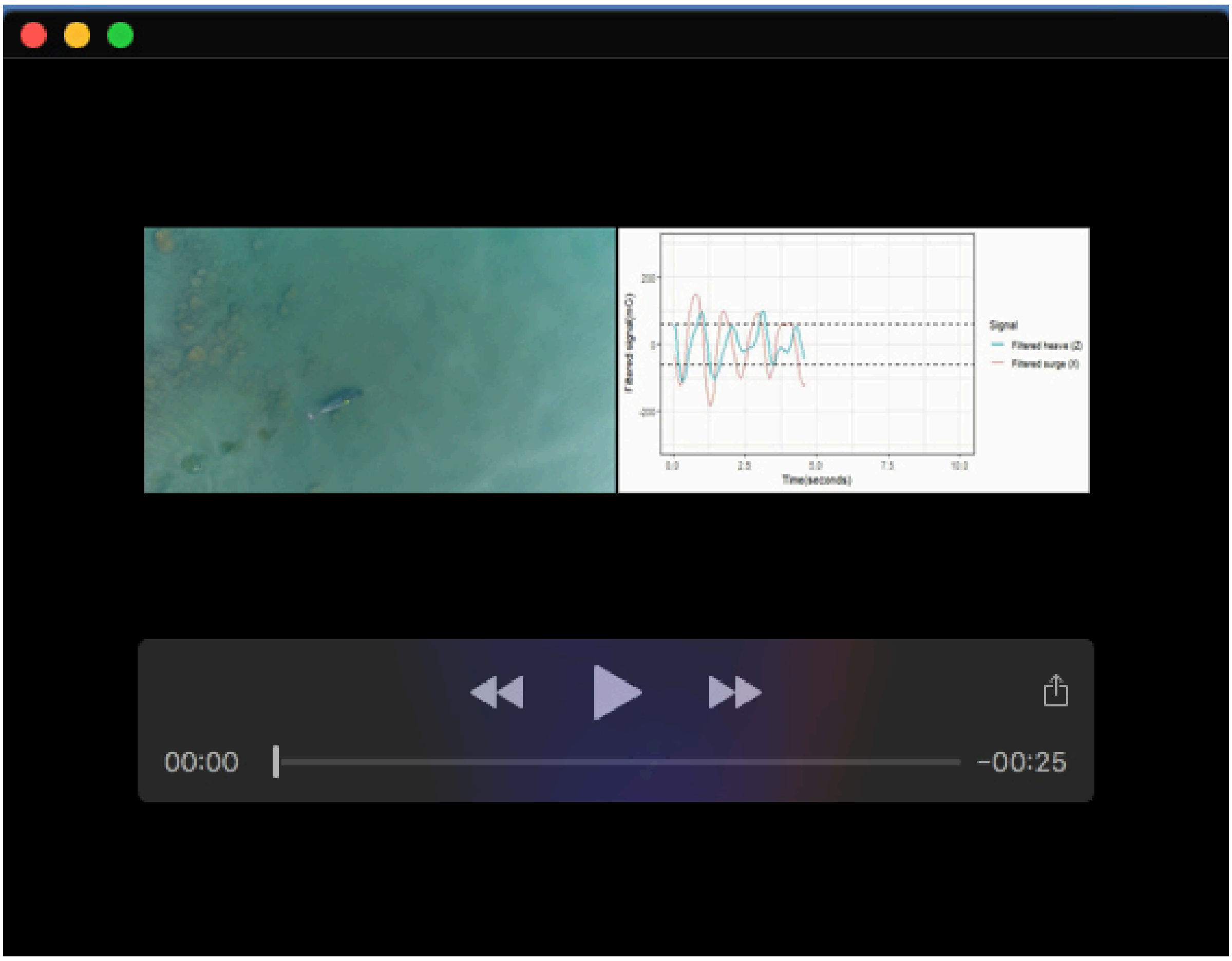

Movie 1. Drone footage of narwhal 2018MM7 swimming away from shore moments after release, along with simultaneous filtered heave and surge acceleration oscillations. Dotted lines indicate the minimum stroking amplitude threshold.
